# Learning environment assessments of a single curriculum being taught at two medical schools 10,000 miles apart

**DOI:** 10.1186/s12909-015-0388-0

**Published:** 2015-06-17

**Authors:** Sean Tackett, Robert Shochet, Nicole A. Shilkofski, Jorie Colbert-Getz, Krishna Rampal, Hamidah Abu Bakar, Scott Wright

**Affiliations:** 1 Johns Hopkins University School of Medicine, Baltimore, MD USA; 2 Medical Education Development and Assessment at the University of Utah School of Medicine, Salt Lake City, Utah USA; 3 Perdana University Graduate School of Medicine, Serdang, Malaysia; 4 Faculty of Medicine, Cyberjaya University College of Medical Sciences, Selangor, Malaysia; 5 4940 Eastern Ave, MFL Center Tower Suite 2300, Baltimore, MD 21224 USA

**Keywords:** Learning environment, Curriculum assessment, International partnerships

## Abstract

**Background:**

Perdana University Graduate School of Medicine (PUGSOM), the first graduate-entry medical school in Malaysia, was established in 2011 in collaboration with Johns Hopkins University School of Medicine (JHUSOM), an American medical school. This study compared learning environments (LE) at these two schools, which shared the same overarching curriculum, along with a comparator Malaysian medical school, Cyberjaya University College of Medical Sciences (CUCMS). As a secondary aim, we compared 2 LE assessment tools - the widely-used Dundee Ready Educational Environment Measure (DREEM) and the newer Johns Hopkins Learning Environment Scale (JHLES).

**Methods:**

Students responded anonymously at the end of their first year of medical school to surveys which included DREEM, JHLES, single-item global LE assessment variables, and demographics questions.

**Results:**

Respondents included 24/24 (100 %) students at PUGSOM, 100/120 (83 %) at JHUSOM, and 79/83 (95 %) at CUCMS. PUGSOM had the highest overall LE ratings (p < 0.05) [DREEM 155.3 (SD 21.3); JHLES 116.5 (SD 12.2)], followed by JHUSOM [DREEM 143.3 (SD 22.5); JHLES 111.7 (SD 12.0)] and CUCMS [DREEM 138.5 (SD 22.4); JHLES 106.4 (SD 14.5)]. PUGSOM’s overall high LE ratings were driven by responses in “perception of teaching,” “meaningful engagement,” and “acceptance and safety” domains. JHLES detected significant differences across schools in 5/7 domains and had stronger correlations than DREEM to each global LE assessment variable.

**Conclusions:**

The inaugural class of medical students at PUGSOM rated their LE exceptionally highly, providing evidence that transporting a medical school curriculum may be successful. The JHLES showed promise as a LE assessment tool for use in international settings.

## Background

American academic health centers (AHC) are expanding their activities internationally to diversify and advance their missions in clinical care, research, and education [1, 2]. An ambitious example of this is for an American medical school to start a new medical school in another country. Weill Cornell Medical College in Qatar (WCMC-Q) was the first such venture in 2001 [3], followed by Duke University – National University of Singapore (NUS) Graduate Medical School in Singapore several years later [4]. On November 2, 2010, Johns Hopkins entered into an agreement to create an AHC in Malaysia, where it would work with partners to develop education, research, and clinical facilities in Malaysia’s first graduate-entry medical school: Perdana University Graduate School of Medicine (PUGSOM), which matriculated its first class less than 1 year later. While such enterprising international partnerships can reap strategic mutual benefits, previously reported experiences have described unanticipated challenges in medical education partnerships, particularly in sensitive content areas like cultural competence [5] and ethics [6].

The medical school learning environment, which “encompasses the physical, social, and psychological context in which students learn; all interactions with faculty, staff, and peers; and the formal, informal, and hidden curricula,” [7] is being increasingly recognized as crucial to medical student development and maturation [8–13]. When medical curricula are developed in one context and implemented in another, it is difficult to predict whether a positive learning environment will be created, making empiric learning environment assessment especially important in these situations.

Assessing the character and quality of the medical school learning environment remains challenging. There are at least 15 published instruments to assess undergraduate medical school learning environments, but none provide strong validity evidence [14]. The most widely-used learning environment assessment tool is the Dundee Ready Educational Environment Measure (DREEM) [14, 15]; however the DREEM was not initially grounded in learning theory, its five subscales have not been empirically validated [16], and it may be dated, having been created in the 1990s. Moreover, DREEM has not been used in American medical schools. A new learning environment assessment tool, called the Johns Hopkins Learning Environment Scale (JHLES), displayed promising psychometric properties in its validation study at JHUSOM [17] and may be useful if applied abroad.

The primary goal of this study was to determine whether a high quality learning environment was established during the first year of the implementation of the JHUSOM curriculum at PUGSOM. To do this, we compared students’ assessments of their learning environments at PUGSOM to those of students at JHUSOM, and those at Cyberjaya University College of Medical Science (CUCMS), a medical school established in the M.B.B.S. model in Malaysia. We used both DREEM and JHLES to enhance the validity of our learning environment assessments, and we compared the performance of JHLES and DREEM at the 3 medical schools.

## Method

### Study design and data collection

This was a cross-sectional survey given to all first year medical students at 3 medical schools at the end of the 2011–2012 academic year. Surveys were administered electronically at PUGSOM and JHUSOM and given on paper, using optical mark reader sheets, at CUCMS. Participation was voluntary, informed consent was obtained from each participant (all of whom were adults), responses were anonymous, and data were de-identified and analyzed by one author who had no role in teaching or evaluating medical students at any of the schools. IRB approval for the complete study protocol was obtained from Johns Hopkins Medicine (NA_00075019 and NA_00065786) and Perdana University (NA_00071425).

### Subjects and setting

JHUSOM was founded in Baltimore, MD in 1893 and follows the graduate-entry model (i.e. all matriculating students must have a college degree). The most recent major curriculum revision created the Genes-to-Society curriculum [18], which was implemented for the 2009 entering class. Students experience a mix of educational methods, and peer-learning is emphasized. Students are very active in their learning communities, in student groups, and in other extracurricular activities. A new medical education building opened in 2009.

PUGSOM is a graduate-entry medical school established as a public-private partnership between Chase Perdana and Johns Hopkins. Its first cohort of students was selected in the spring of 2011 and matriculated in September 2011 to an interim campus near the site of the future medical school campus, about 70 km outside of Kuala Lumpur, Malaysia. Of the students in the first cohort at PUGSOM, all are Malaysian citizens, with the exception of one international student. The overarching Genes-to-Society curriculum was in place and taught by many of the same faculty who taught at JHUSOM in Baltimore.

CUCMS is a private medical school located in Cyberjaya, Malaysia. CUCMS was founded in 2005, and its first class matriculated in December 2005. It follows the school-leaver model of medical education (i.e. students enroll in a 5-year medical program after completing a one-year pre-medical matriculation program (post-high school), A-level certificate, or their equivalent. In the first year, students engage in “student-centered team-based learning,” intended to foster interaction and peer-learning. In this model, the class is subdivided into small groups of 10–12 students, groups which are consistent through all activities. Pedagogy is a mix of interactive lectures and problem-based learning.

### Survey composition

The survey was developed by our study team from institutions in the U.S. and Malaysia, who have extensive experience in medical education and educational research. The survey included the DREEM [19], JHLES [17], several questions related to an overall assessment of the learning environment, and demographics questions.

The DREEM is a 50-item survey where students respond with their level of agreement across a 5-point scale. Items were grouped by its developers into 5 categories: (1) perception of teachers, (2) perception of teaching, (3) academic self-perception, (4) perception of atmosphere, and (5) social self-perception. Each item is scored 0–4 so that composite DREEM scores can range from 0–200, with higher scores indicating a more positive perception of the learning environment.

The JHLES was developed over a series of steps from 2010 to 2012 at JHUSOM [17]. It has 28 items each with 5-point response options. During development, exploratory factor analysis resulted in 7 domains: (1) community of peers, (2) faculty relationships, (3) academic climate, (4) meaningful engagement, (5) mentorship, (6) acceptance and safety, and (7) physical space. Each item is scored 1–5 so that JHLES totals can range from 28 to 140, with higher scores indicating a more positive perception of the learning environment.

We included 3 single-item global learning environment assessment variables. We asked students to rate (i) their overall perception of the learning environment as either (1) terrible, (2) poor, (3) fair, (4) good or (5) exceptional and to rate their agreement with 2 statements on a 1 (strongly disagree) to 5 (strongly agree) scale: (ii) “The overall quality of the educational experience at the School of Medicine is excellent,” and (iii) “Based on my sense of the learning environment at the School of Medicine, I would recommend it to a close friend as a great school to attend”.

### Data analysis

Basic descriptive statistics were tabulated with tests for significant differences applied as appropriate. Overall DREEM and JHLES scores were computed by summing across survey items for each tool. Domain scores were computed by averaging item scores in each domain.

DREEM and JHLES total scores and domain scores were compared across schools, with one-way ANOVA and Kruskall-Wallis tests where appropriate. For institutional pairwise comparisons, we used t-tests with Bonferroni correction.

Pearson correlation coefficients were calculated to determine associations between each of the scales and their respective subcategories or domains. Spearman correlation coefficients were calculated for associations between learning environment scale totals and global LE assessment variables. Areas-under-the-curve were calculated for receiver-operating-characteristic curves created using JHLES and DREEM scale totals as a predictor of the most favorable response (e.g. “strongly agree”) for each global assessment variable.

Stata 13 (StataCorp. 2013. Stata Statistical Software: Release 13. College Station, TX: StataCorp LP.) was used for data analysis.

## Results

We obtained complete surveys from 100/120 (83 %) students at JHUSOM, 24/24 (100 %) students at PUGSOM, and 79/83 (95 %) students at CUCMS (Table 1). Average ages were 23.8 years (SD 2.0) at JHUSOM, 25.3 years (SD 1.9) at PUGSOM, and 20.6 years (SD 1.2) at CUCMS, significantly different at p < 0.01 for all comparisons. Men numbered 50 (50 %) at JHUSOM, 7 (29 %) at PUGSOM, and 34 (43 %) at CUCMS (p = 0.196). Ethnic make-up varied considerably across sites: at JHUSOM most students were white (54 %), at PUGSOM half were Chinese (50 %), and at CUCMS most were Malay (83 %).

**Table 1:** Characteristics of medical schools and respondents at the 3 study sites

		JHUSOM		PUGSOM		CUCMS	
School Characteristics	Location	Baltimore, Maryland, USA		Serdang, Selangor, Malaysia		Cyberjaya, Selangor, Malaysia	
	Year of first students	1893		2011		2005	
	Program model	M.D.		M.D.		M.B.B.S.	
		Graduate-entry		Graduate-entry		School-leaver entry	
	Private/public	Private		Public-private		Private	
	Curriculum	Genes-to-Society, started 2009		Genes-to-Society, started 2011		Student-centered team-based learning, started 2005	
	Buildings	New 2009		Interim campus while permanent campus is constructed		New 2005	
Respondent Characteristics	Response rate	100/120 (83 %)		24/24 (100 %)		79/83 (95 %)	
	Age in years, mean (SD)^a^	23.8 (2.0)		25.3 (1.9)		20.6 (1.2)	
	Male, n (%)^b^	50 (50 %)		7 (29 %)		34 (43 %)	
	Ethnicity, n (%)	White	54 (54 %)	Malay	3 (12 %)	Malay	69 (83 %)
		Asian	38 (38 %)	Indian	6 (25 %)	Indian	12 (15 %)
		Black	10 (10 %)	Chinese	12 (50 %)	Chinese	1 (1 %)
		Other	2 (2 %)	Other	3 (12 %)	Other	1 (1 %)

^a^Differences in age were significantly different at p < 0.01

^b^Differences in sex were not different, p = 0.196

### Assessment of learning environments across schools using DREEM and JHLES

#### DREEM and JHLES totals

On both DREEM and JHLES, the overall learning environment ratings were highest at PUGSOM [DREEM 155.3 (SD 21.3); JHLES 116.5 (SD 12.2)], followed by JHUSOM [DREEM 143.3 (SD 22.5); JHLES 111.7 (SD 12.0)] and then CUCMS [DREEM 138.5 (SD 22.4); JHLES 106.4 (SD 14.5)] (Table 2). There were no significant differences in DREEM or JHLES total scores by age, gender, or ethnicity, when controlling for medical school.

**Table 2:** DREEM and JHLES total and domain scores from the students at the 3 medical schools studied

		PUGSOM (P)	JHUSOM (J)	CUCMS (C)	*p* value^a^	Pairwise *p* values^b^	
DREEM, mean (SD)	Total score	155.3 (21.3)	143.3 (22.5)	138.5 (22.4)	.**010**	**P vs. J**	**.017**
						**P vs. C**	**.002**
						J vs. C	.202
	Teaching	3.3 (0.5)	2.6 (0.6)	2.9 (0.5)	**<.001**	**P vs. J**	**<.001**
						**P vs. C**	**<.001**
						**J vs. C**	**<.001**
	Teachers	3.4 (0.3)	3.2 (0.4)	2.7 (0.4)	**<.001**	P vs. J	.127
						**P vs. C**	**<.001**
						**J vs. C**	**<.001**
	Academic	2.9 (0.5)	2.7 (0.7)	2.8 (0.6)	.333	P vs. J	.130
						P vs. C	.235
						J vs. C	.500
	Atmosphere	2.9 (0.7)	3.0 (0.5)	2.8 (0.7)	.255	P vs. J	.824
						P vs. C	.402
						J vs. C	.080
	Social	2.8 (0.7)	2.8 (0.6)	2.8 (0.5)	.706	P vs. J	.752
						P vs. C	.928
						J vs. C	.709
JHLES, mean (SD)	Total score	116.5 (12.2)	111.7 (12.0)	106.4 (14.5)	.**002**	P vs. J	.072
						**P vs. C**	**.003**
						**J vs. C**	**.009**
	Peers	4.0 (0.7)	4.1 (0.7)	3.9 (0.5)	.021	P vs. J	.615
						P vs. C	.380
						J vs. C	.045
	Faculty	4.4 (0.5)	4.3 (0.5)	3.8 (0.7)	**<.001**	P vs. J	.160
						**P vs. C**	**<.001**
						**J vs. C**	**<.001**
	Academic	4.0 (0.6)	3.6 (0.6)	3.8 (0.6)	**.005**	**P vs. J**	**<.001**
						P vs. C	.197
						J vs. C	.010
	Engagement	4.3 (0.5)	3.7 (0.7)	3.8 (0.7)	.**001**	**P vs. J**	**<.001**
						**P vs. C**	**<.001**
						J vs. C	.709
	Mentorship	3.5 (1.0)	3.7 (0.9)	3.7 (0.9)	.693	P vs. J	.426
						P vs. C	.430
						J vs. C	.966
	Acceptance/Safety	4.6 (0.4)	4.1 (0.6)	3.5 (0.9)	**<.001**	**P vs. J**	**.001**
						**P vs. C**	**<.001**
						**J vs. C**	**<.001**
	Physical Space	3.9 (0.6)	4.4 (0.6)	4.0 (0.7)	**<.001**	P vs. J	.002
						P vs. C	.561
						**J vs. C**	**<.001**

DREEM and JHLES total scores are presented as a sum of all items. Domain averages are presented as average response per item. For DREEM, items are scored 0–4. For JHLES items are scored 1–5

^a^Unadjusted *p* values are presented for Kruskall-Wallis tests across all schools

^b^Unadjusted *p* values are presented for t-tests for pairwise comparisons

Bold values are significant at p < 0.05 after Bonferroni correction

#### DREEM and JHLES subscale and individual items

Ratings differed significantly in 2 of the 5 DREEM categories, for which PUGSOM gave the highest ratings for the “perception of teachers” and the “perception of teaching” categories (Table 2). Overall 15/50 (30 %) DREEM items detected statistically significant differences between schools (p < 0.05 after Bonferroni correction) (Table 3). PUGSOM students responded more favorably than JHUSOM and CUCMS on every item in DREEM’s “perception of teaching” category, with 4/12 (33 %) reaching statistical significance after adjusting for multiple comparisons.

**Table 3:** Percentage of students responding favorably for items from the DREEM and JHLES that were significantly different between schools

Scale	Category/Domain	Survey item	PUGSOM	JHUSOM	CUCMS
DREEM	Teaching	The teaching time is put to good use.	92 %	55 %	78 %
		The teaching is student centered.	100 %	68 %	86 %
		Long-term learning is emphasized over short term.	79 %	34 %	68 %
		The teaching is too teacher-centered.*	88 %	45 %	62 %
	Teachers	The teachers are good at providing feedback to students.	88 %	46 %	70 %
		The teachers ridicule the students.*	67 %	94 %	25 %
		The teachers get angry in class.*	92 %	98 %	20 %
		The teachers are authoritarian.*	75 %	88 %	19 %
		The teachers are patient with patients.	92 %	89 %	59 %
		The students irritate the teachers.*	54 %	76 %	37 %
	Academic	Much of what I have to learn seems relevant to a career in medicine.	100 %	69 %	85 %
	Atmosphere	The atmosphere is relaxed during lectures.	63 %	85 %	49 %
		The atmosphere is relaxed during seminars and tutorials.	67 %	93 %	49 %
		This school is well timetabled.	58 %	55 %	85 %
		Cheating is a problem in this school.*	92 %	93 %	70 %
JHLES	Faculty	I feel that SOM faculty members have taken the time to get to know me.	92 %	80 %	42 %
		Medical school advisors are readily accessible and interested in students.	79 %	96 %	54 %
	Academic	I feel that course exams and assessments test my knowledge and abilities fairly.	88 %	45 %	81 %
	Engagement	The SOM engages students as meaningful participants.	100 %	79 %	79 %
	Acceptance and Safety	I am concerned that students are mistreated at the SOM.*	92 %	91 %	40 %
		I feel concerned at times for my personal safety at the SOM.*	88 %	75 %	51 %

*Abbreviation*: *SOM* school of medicine

Asterisks denote negatively worded items that were reverse coded. For these items, percentages correspond to students responding “disagree” or “strongly disagree”. For the rest of the items, percentages correspond to “strongly agree” and “agree”. All differences were significant at p < 0.05 after Bonferroni correction for multiple comparisons

Ratings differed significantly in 5 of the 7 (71 %) JHLES domains (Table 2). CUCMS gave the lowest ratings for “faculty relationships” and “acceptance and safety.” PUGSOM gave the highest ratings for “acceptance and safety.” JHUSOM gave the highest ratings for “physical space.” Overall, 6/28 (21 %) JHLES items detected statistically significant differences between schools (p < 0.05 after Bonferroni correction) (Table 3). PUGSOM students responded more favorably than JHUSOM and CUCMS on every item in the JHLES “meaningful engagement” and “acceptance and safety” domains, with 1/4 (25 %) and 2/3 (67 %) items reaching statistical significance, respectively.

### Responses to single-item global learning environment assessment variables

Responses to all global learning environment assessment variables showed large majorities reporting an appreciation of the climate at all schools (Table 4). PUGSOM had 100 % of its students rate the overall learning environment as “exceptional” or “good,” ratings which were significantly higher than those at JHUSOM (p = 0065). PUGSOM had a significantly greater proportion of students (100 %) than JHUSOM (85 %, p = .0001) and CUCMS (81 %, p = .0004) who agreed that the overall quality of their educational experience was excellent. Significantly greater proportions of students at JHUSOM (95 %) than at CUCMS (77 %), p = 0.001, agreed they would recommend their medical school to a friend based on their sense of the learning environment.

**Table 4:** Percentage of students responding favorably for single-item global assessment variables of the learning environment

Question	PUGSOM (P)	JHUSOM (J)	CUCMS (C)	*P* value^c^	Pairwise *p* values^d^	
Please rate your overall perception of the learning environment at SOM.^a^	100 %	89 %	89 %	**.020**	**P vs. J**	**.007**
					P vs. C	.085
					J vs. C	.196
The overall quality of the educational experience at the SOM is excellent.^b^	100 %	85 %	81 %	**<.001**	**P vs. J**	**<.001**
					**P vs. C**	**<.001**
					J vs. C	.691
Based on my sense of the learning environment at the SOM, I would recommend it to a close friend as a great school to attend.^b^	92 %	95 %	77 %	**<.001**	P vs. J	.991
					P vs. C	.021
					**J vs. C**	**<.001**

^a^Percentage of students rating learning environment as “exceptional” or “good”

^b^Percentage of students responding “strongly agree” or “agree”

^c^Unadjusted *p* values are presented for Kruskall-Wallis tests across all schools

^d^Unadjusted *p* values are presented for t-tests for pairwise comparisons

Bold values are significant at p < 0.05 after Bonferroni correction

### Correlations of scales and associations with global learning environment assessment variables

DREEM and JHLES scores were highly correlated with one another overall (r = 0.73), with stronger correlations at PUGSOM (r = 0.80) and CUCMS (r = 0.80) compared to JHUSOM (r = 0.64).

JHLES total score was more highly correlated than DREEM total scores across all schools for each of the 3 global learning environment assessment variables. Correlation coefficients for the total study population for JHLES and DREEM were, respectively: 0.53 vs. 0.44 for overall learning environment quality, 0.64 vs. 0.54 for agreeing that the educational experience is excellent, and 0.63 vs. 0.51 for recommending the school to a friend. For analyses for each of these 3 global assessment variables at each school, in 8 of the 9 comparisons, JHLES was more strongly correlated with the global assessment item than was DREEM.

JHLES total score showed better discrimination than the DREEM for identifying students who were most satisfied with their school’s learning environment, with areas-under-the-curve for JHLES and DREEM respectively being: 0.81 vs. 0.73 for overall learning environment quality, 0.82 vs. 0.79 for agreeing the educational experience is excellent, and 0.84 vs. 0.77 for wanting to recommend the school to a friend.

## Discussion

In comparing learning environment assessments by medical students at the end of their first year in medical school, PUGSOM learners rated the learning environment highest on most measures, with most variables being judged more favorably than its American counterpart and a comparison Malaysian school. We also found JHLES scores to be strongly correlated with DREEM scores and our single-item global learning environment assessment variables. JHLES domains successfully detected significant differences between the learning environments across institutions.

Overall ratings of medical school learning environments by students in this study were more favorable than those seen in other studies. Most often, DREEM scores average between 100 and 130 [15, 20], and we were unable to find any published DREEM score from medical schools higher than 143 [21]. Previous studies using DREEM in Malaysia have reported DREEM total mean scores of 117–133 [20, 22, 23]. Most studies report learning environment ratings from senior students or aggregated ratings across student cohorts, but there is some evidence to suggest that first year students may have higher ratings than senior students [20, 24]. It is also known that schools in developed countries, where multiple teaching methods are used (e.g. PBL) and teaching is more interactive, have learning environment ratings that are more positive [15, 25]. It is likely that assessing first year students and more interactive pedagogy can explain in part why all 3 schools scored higher than historical assessments.

To our knowledge, this is the first study to formally assess a medical school learning environment created after a Western academic medical center begins operations in a foreign country. PUGSOM’s exceptional learning environment scores appear to be driven by positive impressions of the teaching methods and faculty and feelings of engagement, acceptance, and safety. Much of this may be related to PUGSOM’s small first year class size, which allows all 24 students to attend all non-clinical teaching sessions together, permitting ample opportunity for interaction with PUGSOM’s small group of core faculty.

Notably, while the curricular content, instructional formats, and even specific faculty were nearly the same at PUGSOM and JHUSOM, PUGSOM responded more positively than JHUSOM on every item in the DREEM “perception of teaching” category, many of which relate to active and student-centered approaches to teaching. Nearly every PUGSOM student completed their pre-medical education in Malaysia, where learning is more teacher-centric and passive, whereas Western teaching styles emphasize learner autonomy and involvement through discussions. PUGSOM’s higher ratings for such elements may therefore represent a greater appreciation for these methods than that of JHUSOM students, who are accustomed to Western teaching styles from their pre-medical education. While this effect may also be related to small class size, the facts that JHUSOM’s students rated their advising higher than PUGSOM’s and the lack of differences in both JHLES’ “community of peers” domain and DREEM’s “social self-perception” category suggest that small class size is not the only factor at play.

The strong correlation between DREEM and JHLES suggests that both surveys may measure the same overall construct, the learning environment. Because many of the individual items from these scales address different areas, one could conjecture that these 2 scales may be complementary. For example DREEM’s questions in the “perception of teaching” and “perception of teachers” categories do not have obvious analogues in the JHLES, primarily because such questions were not found to be informative or were eliminated by factor analysis during JHLES development at JHUSOM. DREEM, on the other hand, does not contain questions that capture student perceptions of their level of engagement, mentorship, or impressions of physical space, which were found to be relevant at JHUSOM and appeared to be important in this study.

JHLES appears to be better able to tease out more differences in perceptions about the learning environment than DREEM. A greater proportion of JHLES domains than DREEM categories revealed differences, and JHLES was more strongly correlated with the 3 distinct global assessments of medical school learning environments. While one could argue that it may be ideal to administer both DREEM and JHLES, the combined 78-item questionnaire this would require may be burdensome. Moreover, a recent confirmatory factor analysis performed on the DREEM, at one medical school in Malaysia [26], suggested 33 DREEM items do not add meaningfully to its learning environment assessment. That the JHLES could discriminate among different factors in 28 items, suggests it may be the preferable instrument, provided it proves to be valid during confirmatory factor analysis on international populations.

Several limitations of this study should be considered. First, PUGSOM was a new school and Malaysia’s only graduate-entry medical school. It had created a new pathway for students to attend medical school, including those who may not have qualified for the traditional M.B.B.S. pathway. Students’ ratings may therefore be influenced by an appreciation for having the opportunity to attend medical school. While we were unable to adjust for this, one may expect that after enduring the rigor of the first year of medical school, the impact of the gratitude as a source of bias might begin to wane. Second, the small class size and the high faculty-student ratios at PUGSOM may play a role in their learning environment perception, although these students did not rate mentoring relationships higher than students at the other schools. Finally, we were not able to collect student grades or other objective educational outcomes, and thus it remains unknown whether learning environment perceptions correspond to meaningful academic success or more empathic patient care.

## Conclusions

Ultimately, this study provides the first empiric evidence that a positive learning environment can be established when a curriculum and teaching style are implemented in a foreign country and within a different culture. Our findings suggest that the learning environment at the international school may even be more positive than its American partner and that of a domestic institution. In future work, it will be necessary to assess changes in the learning environment over time, to examine the factors most responsible for differences perceived in the learning environments, and to ensure that positive learning environments lead to improved professional development and clinical competence.
